# Managers’ perceptions on the implementation of community-based rehabilitation in KwaZulu-Natal

**DOI:** 10.4102/sajp.v80i1.1965

**Published:** 2024-01-26

**Authors:** Sithembiso Blose, Verusia Chetty, Saul Cobbing, Nomzamo Chemane

**Affiliations:** 1 Department of Physiotherapy, Faculty of Health Sciences, University of KwaZulu-Natal, Durban, South Africa; 2 Department of Physiotherapy, Faculty of Health Sciences, University of Toronto, Toronto, Canada

**Keywords:** community-based rehabilitation, rehabilitation, disability, stakeholders, CBR managers, collaboration, people with disabilities, CBR workers

## Abstract

**Background:**

Community-based rehabilitation (CBR) is a World Health Organization (WHO) strategy for social inclusion, equalisation of opportunities and provision of essential services for people with disabilities (PWDs). Community-based rehabilitation is a multi-sectoral strategy that requires all stakeholders to participate equally in its implementation. KwaZulu-Natal has implemented CBR for over two decades, with various stakeholders at the forefront of implementation. However, the status of stakeholder engagement, collaboration and coordination is unknown.

**Objective:**

The objective of our study was to understand how CBR is implemented in KwaZulu-Natal and the roles of each stakeholder in its implementation, with a focus, on managers from government and non-governmental organisations.

**Method:**

A descriptive explorative approach using semi-structured interviews was used to collect data from 20 managers from various stakeholders involved in implementing CBR in KwaZulu-Natal. Data were transcribed and analysed using thematic analysis.

**Results:**

The findings revealed five dominant themes: (1) the understanding of concepts, (2) missed opportunities for implementing CBR, (3) barriers to implementing CBR, (4) benefits to implementing CBR and (5) recommendations for future implementation.

**Conclusion:**

A formal management structure with clear roles and responsibilities was fundamental for implementation. Collaboration, coordination and planning were believed to be the critical roles of managers in the implementation of CBR. Training, awareness and sharing of resources among stakeholders were also identified as important factors in implementing CBR in KwaZulu-Natal.

**Clinical implications:**

Our study will assist managers and clinicians to improve their planning and implementation of CBR.

## Introduction

Community-based rehabilitation (CBR) is a World Health Organization (WHO) strategy that was developed following the Alma Ata declaration, with the aim of improving access to health and rehabilitation services for people with disabilities (PWDs) (WHO 2010). Community-based rehabilitation is a strategy that requires multidisciplinary and inter-sectoral collaboration between various stakeholders for effective and efficient implementation (WHO 2010, 2015). These stakeholders include PWDs; their families; non-governmental organisation (NGOs); civil society, and various government departments at different levels of the state (IDDC 2012; WHO 2015). While CBR is a strategy aimed at improving the quality of life for PWDs, ensuring social inclusion, and improving the equalisation of opportunities, the role of different stakeholders needs to be clearly defined (IDDC 2012). The CBR was initially developed as a strategy for health and rehabilitation services until the creation of the CBR matrix, which was formed following the United Nations Convention on the Rights of Persons with Disabilities (UNCRPD), which outlines key objectives that require a strong partnership between stakeholders (Higashida, Kumara & Gamini Illangasingha 2015; WHO 2010).

The CBR matrix has promoted a shift from the health and rehabilitation focus of CBR to include elements of human rights. The matrix consists of five key pillars: health, education, social, livelihoods and empowerment (WHO 2010). A certain level of planning, co-ordination and integration becomes critical for effective implementation. The relationship between stakeholders is a crucial component of a successful CBR programme.

Accountability, mutual respect and interdependency are the basic, but crucial, elements of an effective programme (Rule et al. 2019). In this, a common understanding of the CBR concepts is vital for all stakeholders. While some have regarded CBR as a complex concept, an in-depth understanding allows for proper multisectoral collaboration and improves the sharing of limited, readily available resources (Rule et al. 2019).

The CBR is aimed at providing rehabilitation services at a community level, leveraging local resources, and using ‘low-tech’ that is less expensive, but is supported by experts and specialists (Jansen-Van Vuuren & Aldersey 2018; Lightfoot 2004). The implementation of CBR requires planning and co-ordination between stakeholders for the effective use of available resources. The lack of a coordinating structure is most often the cause of poor and incorrect implementation of CBR (Soji, Kumar & Varughese 2016). Sharing available resources is the fundamental principle for a successful implementation of any programme (Soji et al. 2016). The resources required include human, transport and financial. The human resources required are usually volunteers, known as community-based rehabilitation workers (CBRW), who are often PWDs, or parents of children with disabilities (Soji et al. 2016). The CBRWs are often linked with local NGOs or Disabled people organizations (DPOs). Various government departments guide the training for CBRWs to provide services that meet the requirements and the identified needs of PWDs. It goes without saying, that the financial aspect of any CBR programme becomes a critical element in the successful implementation and requires careful planning and sharing from different, and involved, government departments (Soji et al. 2016).

The Office on the Status of Persons with Disabilities (OSPD) was established after the post-apartheid era within the Presidency in South Africa (Soji et al. 2016). The main responsibility of OSPD was to monitor the implementation of the Integrated National Disability Strategy (INDS) in all government departments. This role was extended to the Office of the Premier in all provinces. The office of the Premier is responsible for the coordination, integration and promotion of intergovernmental and stakeholder relations (Soji et al. 2016; South African Department of Health 2000). In the KwaZulu-Natal (KZN) office of the Premier, the OSPD is placed within the human rights directorate. One of the crucial mandates of KZN-OSPD is to coordinate and promote stakeholder collaboration and engagement, which includes government departments and NGOs for implementing CBR (Rule, Lorenzo & Wolmarans 2004). However, this office in KZN has encountered numerous challenges in achieving its mandate. The coordination and planning require structures to be available at local, district, provincial and national levels (Soji et al. 2016). Similar to other provinces, KZN has no structures available to plan, coordinate and ensure the implementation of CBR. The CBR implementation has remained the responsibility of each government department’s discretion. The KZN Department of Health (KZN-DoH) has been leading in the implementation of CBR together with a few NGOs. However, the understanding of various managers of how CBR is implemented in KZN and the roles of each stakeholder in the implementation is unknown. Therefore, the aim of our study was to explore the perceptions of various stakeholders on the management of CBR, and its implementation, as well as the role of each stakeholder.

## Methods

An explorative approach was used to understand the perceptions of managers from government and NGOs who are involved in the implementation of CBR in KZN. This approach allowed for the exploration of their experiences and understanding of CBR, as it is currently implemented across the province of KZN, as well as their perceived roles. All data collection was conducted in various offices of the KZN-DoH and the offices of KZN NGOs. The provincial DoH managers, together with 6 out of 11 district programme managers, participated in our study. Three districts are classified as rural, while two are classified as peri-urban, and one is classified as urban. The NGOs involved in our study have their offices situated in urban districts but their work on CBR is in all districts.

Our study used a purposive sampling strategy. The managers from the government and NGOs were selected based on their involvement in CBR programmes in KZN. Recruitment was performed by a research assistant via email, and a telephone call was made as a follow-up to set up appointments with each participant.

### Data collection

Semi-structured interviews were conducted by the first author, using questions developed by the research team using available literature (Grandisson et al. 2014; Pierdomenico & Missionario 2008; Soji et al. 2016). No pilot study was conducted. The use of open-ended questions allowed for probing and clarification to gain a greater understanding of comments and responses during the interview. A total of 26 interviews were conducted. These included six follow-up interviews that were conducted with the same participants from district offices for clarification on some aspects of the initial interviews. All data collection was performed through face-to-face meetings at the participant’s convenient place or area of work. The data were collected using an audio recorder, and notes were also taken during the interview. On an average, the interviews were 45–60 min in duration. All interviews were conducted in English.

### Data analysis

The audio recordings were transcribed verbatim immediately after each interview. The transcriptions were checked for any missing data against the audio recordings by the author and an independent moderator to improve trustworthiness. Data were read and re-read for familiarisation by two of the authors to obtain an in-depth understanding of their content, and thereafter emerging themes and sub-themes were identified. These themes were discussed at length by both authors until consensus was reached. Significant quotes and patterns were coded and moderated separately by an expert in qualitative research approaches. Member checking was performed by returning transcripts to some participants to validate the transcription and interpretation to ensure that the participants agreed with the authors’ interpretation of the emerging themes.

All authors are qualified physiotherapists with experience of more than 20 years in the field of physiotherapy and have worked in both the public and private health sectors.

### Ethical considerations

The ethical approval for our study was obtained from the Biomedical Research Ethics Committee (BREC/00001486/2020) of the University of KwaZulu-Natal. Permission was also granted by the Department of Health Ethics Committee (KZ_202006_039). Pseudonyms are used in the discussion of our study findings to maintain the anonymity of participants.

## Results

The interviews explored managers’ perceptions of the implementation of CBR in KZN. Participants in our study included managers from the Office of the Premier (KZN), the KZN-DoH managers at provincial and district levels and managers from various NGOs rendering CBR services in KZN. Managers from the DoH included physiotherapists, occupational therapists and professional nurses who occupy coordinator posts at district levels.

A total of 20 participants made up of 9 males and 11 females took part: 10 participants were from NGOs and other 10 were from government departments. There were seven PWDs, while 13 were non-disabled. The participants from government departments had experience ranging between 7 years and 19 years, while participants from NGOs ranged between 1 and 30 years in the disability field. All participants had a post-school qualification.

The interviews with managers yielded five themes and sub-themes, namely the understanding of concepts, missed opportunities for implementing CBR, barriers to implementing CBR, benefits of CBR implementation, and recommendations for implementing CBR.

### Understanding of concepts

This theme refers to one’s educational background’s influence in understanding and interpreting CBR. Common understanding and defining key terms or concepts in implementing CBR are important for effective implementation. We found that the background and experiences of participants influenced their definition of CBR.

There was a clear difference in the definition of CBR provided by managers from the DoH and those from NGOs. Participants with disabilities and those who have worked closely with PWDs defined CBR as:

‘A strategy towards the equalisation of opportunities for persons with disabilities and a strategy that works within the community. A strategy that pulls the resources of the community towards the goal of ensuring that persons with disabilities are empowered, they access services, quality, of course. And their voice is heard, and they are properly represented in community structures.’ (BB – assistant director)

‘I will say it is a strategy that is in the community by the community for the community that empowers particularly people with disabilities to understand and be able to stand for their rights that help them as well to be included in community activities to have access to things like education, health, to be employed where they qualify to work.’ (MM – CRF)

Healthcare professionals who were nurses and therapists who participated in our study defined CBR based on their training background:

‘Community-based rehabilitation is moving rehab services to the community where they are most needed, and moving away from this level of health is called tertiary health services, down to community-based services, or to people. where there are people, where these services are needed.’ (GG – district coordinator)

Further to defining CBR, participants, during the interviews, defined rehabilitation and used that definition as a ‘building block’ to unpack their understanding of CBR. Their voices are reflected in the following quotes:

‘Rehabilitation is to assist a person to go back to functionality or near functionality. Be it physical disability, as an example; a person is assisted by therapists and even through CBR to accept his or her condition and to also attain the best functionality level.’ (II – district coordinator)

‘Rehabilitation means a holistic way of looking at disability by looking at the community at large and ensuring that services that are related to either person with disabilities and their families are being accessed according to the constitution and also ensuring rehab also looks at a holistic implementation of public health with relations to the services that pertains to persons with disabilities and their families.’ (EE – district coordinator)

‘Rehabilitation, according to me, I think, it is the, I like the way isiZulu explained it. IsiZulu says it is ukubuyisela emalungelweni (returning to/of rights), so which means it’s just an effort where you take the person who has lost hope and who has just lost his or her identity and try and rebuild their new identity and make that person to understand and to accept that his/her new identity.’ (AA – assistant director)

The approach to CBR and its understanding has also influenced participants’ roles and professional training. The difference in the defining and understanding of terms is also linked to exposure within the disability sector.

### Missed opportunities for implementing community-based rehabilitation

This theme refers to opportunities that are available during the implementation of CBR but are not utilised to strengthen and effectively implement CBR:

‘What is lacking is data collected from CBR must be analyzed and so they can see the impact of CBR if there is any impact and also to provide us with a situation kind of analysis and tell us what is the situation of a disabled person in the society within the province.’ (PP – former chairperson)

highlighting that there is poor analysis, monitoring and evaluation of CBR while information is available.

Furthermore, another participant indicated that the lack of structure or a structured programme contributes to ineffective monitoring and evaluations:

‘The structures, the CBR structures, in fact, we should be having what we call district CBR committees so that in terms of development, people who are there are able to monitor the service together with the service providers so that these forums would form part of resources for me as well, which are non-existent.’ (BB – assistant director)

The monitoring and evaluation were seen as essential for the effective implementation of CBR. The lack of such evaluation reduces the efficient provision of services:

‘The first thing is we need to do monitoring and evaluation of the project to see where we are, what has been done, what is being done, what needs to be done based on the challenges.’ (CC – assistant director)

The lack of utilisation of information was viewed as a missed opportunity, as this information could be used in research that will assist to inform and guide CBR implementation programmes. Some participants believed that the lack of a standardised model for implementing CBR contributed to delivery deficiencies, as clearly illustrated in the following quote:

‘We may have a lot of theories about this, but then if we can have the research of which models work because we have got a lot of models that are coming in but we need to be able to be informed by research that this model is proving to be working.’ (EE – district coordinator)

Another participant said this:

‘Thorough and proper research is required on CBR. We need people who will sit down and do the proper research and try to implement it, not theorise it.’ (AA – assistant director)

The lack of monitoring, evaluation and research in the current implementation programmes of CBR in KZN is seen as a missed opportunity for effective implementation of CBR.

### Barriers to implementing community-based rehabilitation

The CBR was perceived to be implemented throughout KZN by various stakeholders with varying degrees of success, with participants articulating the challenges to implementations.

The lack of resources, both finances and personnel, was regarded as a major barrier to successful implementation of CBR:

‘The lack of human resources, people who understand what CBR is because it is not easy implementing something you do not understand, and government involvement is limited somehow…’ (LL – programmes manager)

‘The resources create a barrier, as much as we would want to implement CBR, as an organization something says no. The funds say no, you cannot jump to that level.’ (SS – programmes manager)

Participants highlighted poor coordination in the implementation of CBR as a further barrier. Participants indicated that they often work individually as a department or NGO or they would work closely with a funder or funding department:

‘That’s a challenge, that we as government are working in silos. There are CBRs which are organised by Department of Corporate Governance and Traditional Affairs, and some are under health but they don’t know each other. There is no co-ordination … yet all these people are serving the same community.’ (QQ – deputy director)

The level of communication among stakeholders in the implementation of CBR was found to be poor. Participants highlighting poor communication as a significant challenge negatively hampering the implementing of CBR. Some voiced their frustration saying:

‘The communication is terrible. It’s the communication because when I try to communicate with them [*DPO*], you don’t get a positive response.’ (DD – district coordinator)

A lack of training was yet another challenge articulated. Most participants said that there is no training for stakeholders on CBR. Some participants became quite angry during interviews when they spoke about the lack of training and the lack of clarity as to who was responsible for training.

‘The provincial disability programmes have promised to come provide training, but I have given him informal information regarding what is expected of him, but no formal training.’ (II – district coordinator)

‘It’s a challenge. As a district, we have never given them any training. I’m hoping that the NGO responsible for them gives them training.’ (FF – district coordinator)

### Benefits of community-based rehabilitation implementation

Participants agreed that CBR has its benefits where it is currently implemented. Despite the barriers, there was an improvement in the services delivered to PWDs in areas where there are active CBR programmes:

‘There were people, we came to know about their situation, and there were people that were referred because of CBR; some people got wheelchairs, who did not have wheelchairs.’ (PP– former chairperson)

‘We are able to identify cases that are there at home and had no access to rehab services.’ (EE – district coordinator)

The improvement in the accessibility of services was not only seen in services rendered by various government departments, especially rehabilitation; but some participants indicated that CBR has assisted in empowering parents of children with disabilities:

‘We refer them to people or get people to come to the parents to help them and they have now accepted that the child has a disability and knows what kind of disability the child has and can now get the help they need.’ (MM – CRF)

### Recommendations for implementing community-based rehabilitation

There were proposals made by participants for improving the implementation of CBR. Some participants felt that people without disabilities are not aware of disability issues and lack basic fundamental knowledge. Improving CBR awareness among stakeholders and community at large was proposed as one of the key fundamentals that can assist in the implementation of CBR:

‘We need to ensure more awareness, not for disabled people, but those without disabilities.’ (KK – public admin)

Furthermore, the decision maker needs to know what CBR is, as their lack of knowledge affects the implementation of CBR:

‘I would say also awareness creation regarding CBR workers… So if you are in a power position of giving or not giving people money, or not giving them, you should at least know about CBR – what it entails.’ (MM – advocacy officer)

Participants believed that better resource allocation needs to be achieved for the effective implementation of CBR:

‘Resources need to be allocated solely for community-based rehabilitation services: human resource, transport, assistive devices and consumables.’ (GG – district coordinator)

The establishment of a coordinating structure was strongly recommended by most participants. The proposal for the development of such a structure was for the coordinating structure to be available at all levels of CBR implementation:

‘When you properly co-ordinate, if you would have the provincial structures. That will be the province CBR structure that would have some structures at district and local level.’ (EE – district coordinator)

In addition,, the availability of a structure to coordinate services was seen as a step towards involving the use of services:

‘The structures, the CBR structures, in fact we should be having what you call district CBR committees so that, in terms of development, people who are consuming the service, they are part, as well of how the service is developed and monitored and they are able to monitor the service together with the service provider.’ (BB – assistant director)

Moreover, research and innovation were identified as crucial elements that the province, together with all stakeholders including academia, need to constantly focus on. This research will guide the development of different strategies for the implementation of CBR:

‘There need to be constant research to inform the development of CBR and to pick up some gaps so that each and every time we develop new approaches’ (BB – assistant director)

## Discussion

The objective of our study was to understand the implementation of CBR in KwaZulu-Natal. Our study undertook to explore the perceptions of stakeholders from government and NGOs on their knowledge and involvement in the implementation of CBR. The findings of the article will be discussed in relation to the five study themes.

### Understanding of concepts

Understanding CBR as a strategy is important for an effective implementation. The development of CBR was initially aimed at improving the delivery of health and rehabilitation services. This has since expanded into a CBR matrix that incorporated other aspects, such as empowerment and livelihood to promote inclusion and human rights (WHO 2010). Understanding rehabilitation and disability becomes crucial for a clear understanding of CBR. According to the WHO, rehabilitation is defined as ‘a set of interventions designed to optimise functioning and reduce disability in individuals with health conditions, in interaction with their environment’ (Skempes, Stucki & Bickenbach 2015). In simple terms, rehabilitation seeks to help individuals of any age be as independent as possible in everyday activities and enables their participation in education, work, recreation and meaningful life roles, such as taking care of family. This aligns with the goals of CBR that aim to promote and encourage social inclusion and participation of PWDs in their communities. Braathen, Munthali and Grut (2015) state that the understanding of disability, especially by healthcare professionals, determines how services are rendered for PWDs and how they will be treated. There is a strong argument that healthcare providers are largely orientated towards the biomedical models of disability because of their training and thus leading to limited access to services. Therefore, services will mainly be hospital-based, rather than community-based (Grut et al. 2012).

We found that the professionals’ background greatly influences their understanding of rehabilitation in general, and CBR in particular. While participants were able to share their understanding of rehabilitation and CBR, responses leaned more towards their understanding of disability or approach to disability. Healthcare professionals usually use the biomedical model of disability in the rendering of health services, including rehabilitation, to PWDs and therefore indirectly contribute to social exclusion and marginalisation of PWDs (Braathen et al. 2015). Furthermore, CBR is hampered by the biomedical model as it promotes institutional services and is disease based; whereas CBR requires for services to be based in the community and encourages barrier removal for inclusion of PWDs. Clearly, the responses of participants who were healthcare professionals were orientated more towards the biomedical model in defining rehabilitation and CBR. By contrast, participants from NGOs (DPOs), especially those with a disability, responded with definitions that lean more towards a social model of disability, which included a rights-based approach. The difference in understanding and defining the concepts of rehabilitation and CBR has a direct influence on the approach to CBR implementation. These differences can negatively affect the stakeholders’ role in the implementation of CBR (Morita et al. 2013).

### Missed opportunities for implementing community-based rehabilitation

The CBR implementation requires a multisectoral approach (Rule et al. 2019). It is essential that various stakeholders contribute effectively by taking an active role in the implementation. A ‘nexus contract’ between stakeholders is required to guide the implementation of CBR, its monitoring, evaluation and reporting. The ‘contract’ is not based on a signed document but on mutual understanding and relationships (Finkenflügel 2006). The authors found that, while CBR is implemented with various stakeholders acknowledging each other’s presence in the process of implementing CBR, there is a lack of monitoring and evaluation of the CBR implementation. Research indicates that many tools are used globally to monitor and evaluate CBR, but no standardised tool exists (Lukersmith et al. 2013). The Department of Health is one of the leading government institutions, working with various NGOs, in the implementation of CBR. However, the province seems to have no system for reporting, monitoring and evaluation. Reports are submitted regularly, but there is no evidence of engagement to monitor and evaluate the implementation of CBR. The system seems to be dependent on reports developed by funded organisation as a method of reporting on the use of funds, but there is no feedback on the implementation of CBR.

The collection of information for planning and developing services further is critical. The CBR has been reported as being rich in data, but presenting poor evidence on the effectiveness of implementation (Kusuwo et al. 2017). Research, formal or informal, can be used to assist in better planning; implementing; reporting; monitoring and evaluation; and in creating transparency among stakeholders, which may lead to more investment in resources. The lack of involvement of researchers was also viewed as a missed opportunity that would probably have assisted not only with research on implementation and monitoring but also in developing systems and tools for reporting, monitoring and evaluation.

### Barriers to implementing community-based rehabilitation

There was general agreement that, in the current implementation of CBR, there are gaps that exist in the implementation methods and strategies. These gaps have posed challenges that hinder the uptake of CBR. Ayalew et al. (2020) identified poor availability of resources and poor collaboration and co-ordination as key barriers in the implementation of CBR in southern Ethiopia (Ayalew et al. 2020). Similarly, these barriers were identified by managers as gaps that affect the implementation of CBR in the province.

It is a common practice that the organisations that provide resources to support other organisations usually dictate the allocation and use of resources, especially funding (Finkenflügel 2006). These practices are usually driven or derived from the organisation’s eagerness to achieve its goals (Finkenflügel 2006). In our study, the NGOs identified the lack of funding as the main barrier, whereas the managers from our Department of Health identified the lack of human resources (staffing) as their main barrier to implementing CBR. The managers of some organisations further explained that, even the little funding that is received, comes with limitations and cannot be used for something that the funder has not approved. In order for resources, such as funding, to be redirected according to the funded organisation’s priority, the decision makers from the funding organisation need to be aligned with, and re-orientated to, the broader goals of CBR (Ayalew et al. 2020).

Higashida et al. (2015) indicate that one of the contributing factors to poor uptake and development of CBR is poor planning, collaboration and co-ordination among stakeholders. The shortage of resources is further compounded by a lack of co-ordination and poor communication (Higashida et al. 2015). In the implementation of CBR, co-ordination and communication among stakeholders are the key elements that allow for planning and sharing of resources at the local level. Therefore, the lack of co-ordination and poor communication results in a duplication of services and depletion of the limited resources. This leads to poor, or no, CBR implementation and a lack of training of the stakeholders (Fiorati et al. 2018).

Training of personnel is fundamental for any programme or project to succeed (CBR Education and Training for Empowerment [CREATE] 2015). The establishment and implementation of CBR in South Africa were based on three training pilot projects. These projects laid the foundation for CBR implementation through training (CREATE 2015). The participants in our study agreed that, in the current implementation of CBR, training of stakeholders is lacking and remains a challenge. Only one organisation indicated that it was involved in training. However, the training was not directed to stakeholders responsible for implementing CBR, but rather to communities and parents of children with disabilities with the aim of establishing support groups. While all participants acknowledged the importance of training, the person or entity responsible for providing the training remained an unaddressed issue.

### Benefits of community-based rehabilitation implementation

Community-based rehabilitation was established to improve services to PWDs (WHO 2015). Although the concept of CBR has expanded into a matrix, the rights of PWDs are at the forefront. These rights include access to services, which include health, education and social services (Samuel 2015). The CBR has been implemented in KwaZulu-Natal through CBRWs (Chappell & Johannsmeier 2009). We observed that, where CBRWs are available, there is increased access to services. It was noticed that PWDs received appropriate assistive devices timeously and were referred for other services, including education, and for social and disability grants. Participants also found that, with the presence of CBRWs, there was better social integration and participation of PWDs in community activities.

### Recommendations for implementing community-based rehabilitation

An understanding of CBR is fundamental for effective implementation. A study conducted in Korea on awareness of CBR in public health facilities found that awareness of CBR was low and this negatively affected the uptake of rehabilitation (Suk Lee et al. 2011). Improving awareness of CBR can lead to improved understanding and an effective implementation of CBR. Community-based rehabilitation awareness campaigns need to be directed to all stakeholders, especially those with roles that can influence the uptake of CBR. Part of creating awareness should include the training of key stakeholders in CBR, including service beneficiaries and communities (Jansen-Van Vuuren & Aldersey 2018).

For any programme to be successful, allocation of adequate resources is essential. For CBR to be implemented effectively, stakeholders recommended that allocation of resources must be improved. Bongo, Dziruni and Muzenda-Mudavanhu (2018), in their study identified the allocation of resources as one of the fundamental principles for the implementation and sustainability of CBR (Bongo et al. 2018). Our study found that the prioritisation of resources differed among stakeholders. The NGOs required more funding as a resource to aid in better delivery of CBR, while government outlined staffing as a major resource. Therefore, government, local authorities and civil society should work together for continual support, the development of CBR, and the allocation of appropriate resources (Bongo et al. 2018). A consensus among stakeholders on the prioritisation of resources must be reached through consistent communication, collaboration and sharing of the currently available, limited resources.

Managing multiple stakeholders and resources requires proper co-ordination. A study in Vietnam found that the development and maintenance of coordinating structures at various levels is fundamental to implementing CBR programmes (Mijnarends et al. 2011). One of the challenges that our study identified was the poor or lack of coordinating structures.

The establishment of coordinating structures at different levels of service will not only assist with coordinating services but will also improve communication and sharing of resources among stakeholders (Mijnarends et al. 2011). Currently, the Office on the Status of PWDs, in the office of the Premier, is best suited to take a leading role in the coordination.

The planning, managing of resources, monitoring and evaluation of CBR programmes should not be neglected as part of CBR development and implementation. Therefore, relevant structures should be established (Pierdomenico & Missionario 2008). The co-ordination of CBR may require the establishment of structures at different levels of government. These structures will need to have clearly defined roles and responsibilities, together with good communication. The establishment of coordinating structures will also assist with the development, implementation, monitoring and evaluation of the implementation of CBR services.

Different organisations implement CBR using different approaches. Continual research conducted to support CBR implementation and to identify gaps is encouraged (Kusuwo et al. 2017). Participants in our study recommended research and innovation as important in the implementation and monitoring of CBR in the province. Training and research have been conducted by institutions of higher learning (Lorenzo & Motau 2014). However, the recommendations and findings have not reached the relevant levels, or been implemented by the authorities, to effect the necessary changes that will assist with the development and implementation of CBR services in the province (Lukersmith et al. 2013).

Academia can play a role in the development and implementation of CBR in a way that will assist in addressing community needs. Stoecker (2003) indicated that community-based research is necessary to bridge a gap between academia and community through partnership and research. Through gathering and disseminating knowledge, community needs can be identified and addressed collaboratively to provide positive social action for transformation (Stoecker 2003). Similarly, we found that academics can also assist with effective ways and methods to implement CBR. Participants suggested that the institutions of higher learning and academia can assist with providing a clear and understandable definition of CBR in a way that can be understood by all stakeholders. Therefore, academic research can assist with the development of definitions, innovative methods and appropriate strategies for implementation, and provide tools for assessing, planning, monitoring and evaluation.

## Conclusion

The CBR is a complex strategy that requires multiple stakeholders for effective implementation. A mutual understanding of CBR, from definition to implementation and monitoring, is a vital component for the effective delivery of CBR. While the implementation of CBR is faced with many challenges, the collaboration, communication and engagement of stakeholders, from government departments, NGOs or civil society organisations, can assist by minimising the duplication of services, sharing the available limited resources, and planning, monitoring and evaluating CBR implementation.

The development of CBR structures may be achieved effectively by also including roles and responsibilities from a provincial level to a community level. However, the major hurdle that will require proper planning and communication between stakeholders is the issue of financial resources. With proper planning, co-ordination and monitoring, the sharing of this much-needed resource can be achieved.

Research can contribute significantly to the development and implementation of CBR in KZN. Planning, development, implementation, monitoring and evaluation of CBR will be achieved with adequate and proper research, training innovations, and the development of standardised programmes for the implementation of CBR.

## References

[CIT0001] Ayalew, A.T., Adane, D.T., Obolla, S.S., Ludago, T.B., Sona, B.D. & Biewer, G., 2020, ‘From community-based rehabilitation (CBR) services to inclusive development. A study on practice, challenges, and future prospects of CBR in gedeo zone (Southern Ethiopia)’, *Frontiers in Education* 5, 1–17. 10.3389/feduc.2020.506050

[CIT0002] Bongo, P.P., Dziruni, G. & Muzenda-Mudavanhu, C., 2018, ‘The effectiveness of community-based rehabilitation as a strategy for improving quality of life and disaster resilience for children with disability in rural Zimbabwe’, *Jàmbá: Journal of Disaster Risk Studies* 10(1), 1–10. 10.4102/jamba.v10i1.442 PMC6014043 29955257

[CIT0003] Braathen, S.H., Munthali, A. & Grut, L., 2015, ‘Explanatory models for disability: Perspectives of health providers working in Malawi’, *Disability and Society* 30(9), 1382–1396. 10.1080/09687599.2015.1099517

[CIT0004] CBR Education and Training for Empowerment (CREATE), 2015, *Understanding community based rehabilitation in South Africa*, Pietermaritzburg, viewed June 2023, from http://www.create-cbr.co.za/cbrstudy.

[CIT0005] Chappell, P. & Johannsmeier, C., 2009, ‘The impact of community based rehabilitation as implemented by community rehabilitation facilitators on people with disabilities, their families and communities within South Africa’, *Disability and Rehabilitation* 31(1), 7–13. 10.1080/09638280802280429 19194807

[CIT0006] Finkenflügel, H., 2006, ‘Who is in … and for what? An analysis of stakeholders’ influences in CBR’, *Asia Pacific Disability Rehabilitation Journal* 17(1), 12–34.

[CIT0007] Fiorati, R.C., Dakazuka Carretta R.Y., Joaquim, K.P., Ferreira Placeres, A.F. & Jesus, T.S., 2018, ‘Anticipated barriers to implementation of community-based rehabilitation in Ribeirão Preto, Brazil’, *Disability, CBR & Inclusive Development* 29(1), 5–25. 10.5463/dcid.v29i1.701

[CIT0008] Grandisson, M., Thibeault, R., Hébert, M. & Templeton, A., 2014, ‘Community-based rehabilitation programme evaluations: Lessons learned in the field’, *Disability, CBR & Inclusive Development* 25(1), 55. 10.5463/DCID.v25i1.240

[CIT0009] Grut, L., Mji, G., Braathen, S.H. & Benedicte, I., 2012, ‘Accessing community health services: Challenges faced by poor people with disabilities in a rural community in South Africa’, *African Journal of Disability* 1(1), 19. 10.4102/ajod.v1i1.19 28729977 PMC5442570

[CIT0010] Higashida, M., Kumara, M.R.S. & Gamini Illangasingha, M., 2015, ‘Promoting participation of stakeholders in community-based rehabilitation in Sri Lanka: Process of action research in Anuradhapura’, *International Journal of Social Science Studies* 3(3), 50–60. 10.11114/ijsss.v3i3.732

[CIT0011] IDDC, 2012, *CBR guidelines as a tool for community based inclusive development*, International Disability and Development Consortium, Belgium.

[CIT0012] Jansen-Van Vuuren, J.M. & Aldersey, H.M., 2018, ‘Training needs of community-based rehabilitation workers for the effective implementation of CBR programmes’, *Disability, CBR and Inclusive Development* 29(3), 5–31. 10.5463/dcid.v29i3.742

[CIT0013] Kusuwo, P., Myezwa, H., Pilusa, S. & M’kumbuzi, V., 2017, ‘A systematic review to identify system-related elements that can be used to evaluate community-based rehabilitation (CBR) programmes’, *European Journal of Physiotherapy* 19(Suppl. 1), 41–46. 10.1080/21679169.2017.1381323

[CIT0014] Lightfoot, E., 2004, ‘Community-based rehabilitation a rapidly growing method for supporting people with disabilities’, *International Social Work* 47(4), 455–468+559+561+563+565+567. 10.1177/0020872804046253

[CIT0015] Lorenzo, T. & Motau, J., 2014, ‘A transect walk to establish opportunities and challenges for youth with disabilities in Winterveldt, South Africa’, *Disability, CBR & Inclusive Development* 25(3), 45. 10.5463/dcid.v25i3.232

[CIT0016] Lukersmith, S., Hartley, S., Kuipers, P., Madden, R., Llewellyn, G. & Dune, T., 2013, ‘Community-based rehabilitation (CBR) monitoring and evaluation methods and tools: A literature review’, *Disability and Rehabilitation* 35(23), 1941–1953. 10.3109/09638288.2013.770078 23574396

[CIT0017] Mijnarends, D.M., Pham, D., Swaans, K., Van Brakel, W.H. & Wright, E.P., 2011, ‘Sustainability criteria for CBR programmes – Two case studies of provincial programmes in Vietnam’, *Disability, CBR & Inclusive Development* 22(2), 3–21. 10.5463/dcid.v22i2.54

[CIT0018] Morita, H., YasuHara, K., Ogawa, R. & Hatanaka, H., 2013, ‘Factors impeding the advancement of communitybased rehabilitation (CBR): Degree of understanding of professionals about CBR’, *Journal of Physical Therapy Science* 25(4), 413–423. 10.1589/jpts.25.413

[CIT0019] Pierdomenico, L. & Missionario, S., 2008, *Improving CBR management at provincial level community based rehabilitation in Vietnam view project try out to develop CBR understanding and activities in Bac Ninh province Vietnam view project*, viewed June 2023, from https://www.researchgate.net/publication/215748202.

[CIT0020] Rule, S., Lorenzo, T. & Wolmarans, M., 2004, *Community-based rehabilitation: New challenges*, Human Sciences Research Council Press (HSRC Press).

[CIT0021] Rule, S., Roberts, A., McLaren, P. & Philpott, S., 2019, ‘South African stakeholders’ knowledge of community-based rehabilitation’, *African Journal of Disability* 8, 1–12. 10.4102/ajod.v8i0.484. PMC6779963 31616621

[CIT0022] Samuel, J.U., 2015, ‘Utilization of community based rehabilitation for persons’ with disabilities (PWD) in Nigeria: The way forward’, *European Scientific Journal* 11(25), 80–88, viewed June 2023, from https://www.researchgate.net/publication/282333434.

[CIT0023] Skempes, D., Stucki, G. & Bickenbach, J., 2015, ‘Health-related rehabilitation and human rights: Analyzing states’ obligations under the united nations convention on the rights of persons with disabilities’, *Archives of Physical Medicine and Rehabilitation* 96(1), 163–173. 10.1016/j.apmr.2014.07.410 25130185

[CIT0024] Soji, F., Kumar, J. & Varughese, S., 2016, ‘Sustainability: Lessons from a community-based rehabilitation programme in Karnataka, India’, *Knowledge Management for, Development Journal*, viewed June 2023, from http://journal.km4dev.org/.

[CIT0025] South African Department of Health, 2000, *National rehabilitation policy, national rehabilitation policy*, South African Government, Pretoria.

[CIT0026] Stoecker R., 2003, *Principles of best practice for community-based research*, viewed June 2023, from: https://www.researchgate.net/publication/284329269.

[CIT0027] Suk Lee, H., Sung CHang, J., HYun kwon, Y. & CHoi, Y., 2011, ‘Awareness of community-based rehabilitation with a focus on public health centers’, *Journal of Physical Therapy Science* 23(6), 909–913. 10.1589/jpts.23.909

[CIT0028] World Health Organization (WHO), 2010, *CBR guidelines*, World Heath Organization, Geneva.

[CIT0029] World Health Organization (WHO), 2015, *WHO global disability action plan 2014–2021: Better people health with for disability*, WHO Publications, Geneva.

